# Photocatalytic antibacterial performance of TiO_2_ and Ag-doped TiO_2_ against *S. aureus*. *P. aeruginosa* and *E. coli*

**DOI:** 10.3762/bjnano.4.40

**Published:** 2013-06-06

**Authors:** Kiran Gupta, R P Singh, Ashutosh Pandey, Anjana Pandey

**Affiliations:** 1 Department of Chemistry, Motilal Nehru National Institute of Technology, Allahabad-211004. U.P., India. Phone: +91-0532-2271276; 2 Nanotechnology & Molecular Biology Lab, Centre of Biotechnology, University of Allahabad, Allahabad – 211002, U.P., India; 3 Department of Biotechnology, Motilal Nehru National Institute of Technology, Allahabad -211004. U.P., India.

**Keywords:** Ag-doped TiO_2_, antimicrobial activity, sol–gel

## Abstract

This paper reports the structural and optical properties and comparative photocatalytic activity of TiO_2_ and Ag-doped TiO_2_ nanoparticles against different bacterial strains under visible-light irradiation. The TiO_2_ and Ag-doped TiO_2_ photocatalysts were synthesized by acid catalyzed sol–gel technique and characterized by X-ray diffraction (XRD), transmission electron microscopy (TEM), UV–vis spectroscopy and photoluminescence (PL). The XRD pattern revealed that the annealed sample of TiO_2_ has both anatase and rutile phases while only an anatase phase was found in Ag-doped TiO_2_ nanoparticles. The decreased band-gap energy of Ag-doped TiO_2_ nanoparticles in comparison to TiO_2_ nanoparticles was investigated by UV–vis spectroscopy. The rate of recombination and transfer behaviour of the photoexcited electron–hole pairs in the semiconductors was recorded by photoluminescence. The antimicrobial activity of TiO_2_ and Ag-doped TiO_2_ nanoparticles (3% and 7%) was investigated against both gram positive (*Staphylococcus aureus*) and gram negative (*Pseudomonas aeruginosa*, *Escherichia coli*) bacteria. As a result, the viability of all three microorganisms was reduced to zero at 60 mg/30 mL culture in the case of both (3% and 7% doping) concentrations of Ag-doped TiO_2_ nanoparticles. Annealed TiO_2_ showed zero viability at 80 mg/30 mL whereas doped Ag-TiO_2_ 7% showed zero viability at 40 mg/30 mL culture in the case of *P. aeruginosa* only.

## Introduction

The photocatalytic agent TiO_2_, known for its chemical stability and optical competency, has been used extensively for killing different groups of microorganisms including bacteria, fungi and viruses, because it has high photoreactivity, broad-spectrum antibiosis and chemical stability [1–6]. The photocatalytic activity of annealed TiO_2_ sturdily depends upon its existing phase, i.e., anatase, rutile, brokite. The anatase phase shows an indirect optical band gap of 3.2 eV, while the rutile phase has a direct band gap of 3.06 eV and an indirect one of 3.10 eV [7]. However, crude nanoparticles are amorphous in nature, with decreased surface area, and show a fast recombination rate of electrons and holes. Finally the antibacterial activity is decreased. The photocatalytic activity of TiO_2_ nanoparticles depends not only on the properties of the TiO_2_ material itself, but also on the modification of TiO_2_ with metal or metal oxide. Previous studies reported that the addition of noble metal (silver and gold) in titanium dioxide enhances its photocatalytic efficiency [8–9]. However, silver nanoparticles have prospective applications including biosensing, biodiagnostics, optical fibers, and antimicrobial and photocatalytic uses. Silver ions are known to cause denaturation of proteins present in bacterial cell walls and slow down bacterial growth [5]. The simplest photocatalytic mechanism of silver ions is that it may take part in catalytic oxidation reactions between oxygen molecules in the cell and hydrogen atoms of thiol groups, i.e., two thiol groups become covalently bonded to one another through disulfide bonds (R–S–S–R), which leads to blocking of respiration and cell death of the bacteria [10]. Another remarkable mechanism of the antimicrobial activity of Ag nanoparticles is related to the formation of free radicals and consequent free-radical-induced oxidative damage of the cell membranes of bacteria [11–12]. But the same result was not found with gold nanoparticles [12].

Previously, it was observed that doping of a TiO_2_ matrix with silver ions moved the absorption to a longer wavelength, i.e., to the visible region in comparison with pure TiO_2_, due to the change in electronic and optical properties of TiO_2_ [13]. On the other hand, TiO_2_ is also an excellent supporting metal oxide for the doping of silver nanoparticles due to its small crystal size and high surface area.

The aim of this work is to investigate the comparative photocatalytic activity of TiO_2_ and Ag-doped TiO_2_ (visible light active) nanoparticles synthesized by acid-catalyzed sol–gel technique. The prepared particles were characterized by X-ray diffraction (XRD), transmission electron microscopy (TEM), ultraviolet visible spectroscopy (UV–vis) and photoluminescence (PL). Furthermore, the antibacterial activity of the TiO_2_ and Ag-TiO_2_ nanoparticles were investigated against Gram-positive *Staphylococcus aureus (S. aureus)*, and Gram-negative *Pseudomonas aeruginosa* (*P. aeruginosa*) and *Escherichia coli* (*E. coli*) bacteria under visible light.

## Results and Discussion

### XRD of TiO_2_ and Ag-doped TiO_2_

**Figure 1:**
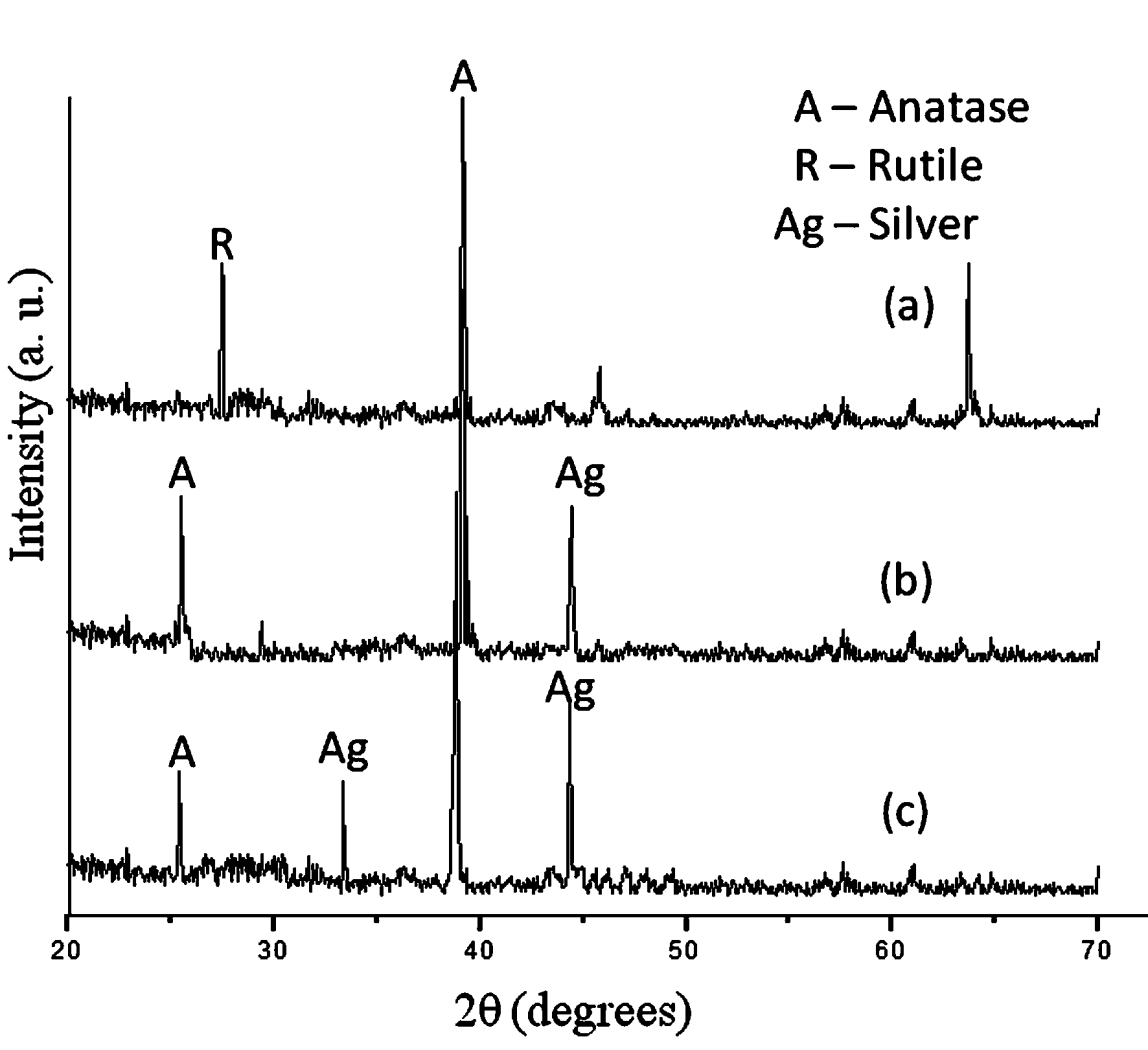
XRD pattern of (a) TiO_2_ and (b) 3% and (c) 7% Ag-doped TiO_2_ nanoparticles annealed at 450 °C.

The samples were annealed at 450 °C to achieve crystallization in TiO_2_ and Ag-doped TiO_2_ nanoparticles. The crystal size of as-prepared TiO_2_ and Ag-doped TiO_2_ nanoparticles were calculated by the Scherrer equation based on the wide-angle XRD as shown in Figure 1. The typical anatase phase was observed in the case of Ag-doped TiO_2_ while in the case of pure annealed TiO_2_ both phases, anatase and rutile, were present. The crystal sizes of the annealed TiO_2_ and Ag-doped TiO_2_ (two different concentrations of AgNO_3_) were 22 nm, 18 nm and 16 nm as calculated by Scherrer equation based on analysis of the wide-angle XRD peak broadening at the (101) peak present in Figure 1. The Ag-doped TiO_2_ nanoparticles clearly exhibit the (200) diffraction peak of the metallic silver and showed a face-centered cubic structure of metallic silver (Figure 1b,c). It was reported that the intensities of the anatase peaks decreased in comparison to rutile peaks as the annealing temperature increased, and after annealing at 800 °C a complete rutile TiO_2_ phase was obtained [14]. A previous study found that a mixture of anatase and rutile TiO_2_ nanoparticles exhibits greater photocatalytic activity than do pure anatase or pure rutile TiO_2_ nanoparticles under excitation by UV light [15]. Furthermore, as reported in a previous study, calcinations of nanoparticles can increase the crystallinity of TiO_2_, which leads to a decrease in the photo-excited e^−^–h^+^ recombination; and thus, increases the photocatalytic activity of TiO_2_ [16].

### TEM analysis

**Figure 2:**
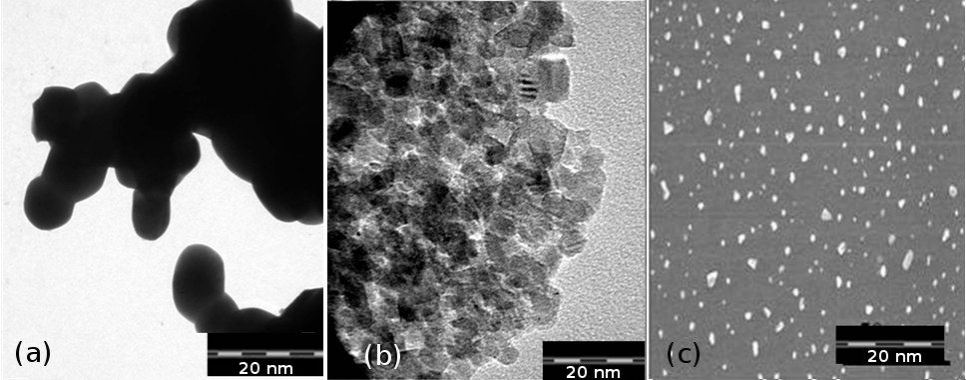
TEM images of (a) TiO_2_ and (b) 3% and (c) 7% Ag-doped TiO_2_ nanoparticles annealed at 450 °C.

The shape and size of undoped and Ag-doped TiO_2_ nanoparticles (3% and 7%) were analyzed by TEM images in Figure 2. The crystal sizes are ≈10 nm, ≈8 nm and ≈5 nm for (a) TiO_2_ and (b) 3% and (c) 7% Ag-doped TiO_2_ nanoparticles, respectively, according to TEM images. The particles are irregular in shape but agglomerated in the case of TiO_2_ (Figure 2a) and Ag-doped TiO_2_ with a 3% concentration of silver salt (Figure 2b), whereas they are scattered in the case of Ag-doped TiO_2_ nanoparticles with a 7% concentration (Figure 2c) of silver salt.

### UV–vis spectroscopy

**Figure 3:**
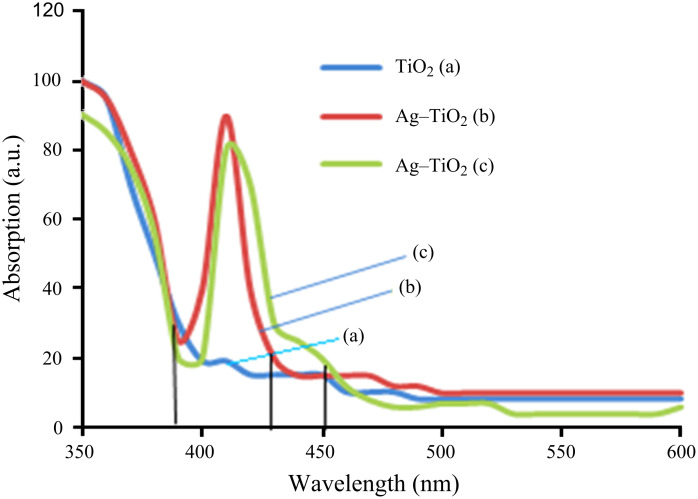
UV–vis absorption spectra of the annealed (a) TiO_2_ and (b) 3% and (c) 7% Ag-doped TiO_2_ nanoparticles.

Optical properties were observed by UV–vis spectroscopy. Figure 3 demonstrates the optical absorption spectra of TiO_2_ and Ag/TiO_2_ (3% and 7%) nanoparticles. The absorption edge of TiO_2_ nanoparticles at 385 nm moved to a longer wavelength after doping with Ag ions (3% and 7%), showing the absorption edge at 435 nm and 450 nm, respectively. After doping with silver ions the response of TiO_2_ nanoparticles to visible light was increased and showed red shift (towards increased wavelength). The red shift of the absorption curve results in a reduction of the band gap energy and also the recombination rate, and hence, enhanced photocatalytic activity.

The optical absorption coefficient α of a semiconductor is expressed by the following equation:


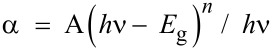


Here α is the absorption coefficient, *E*_g_ is the absorption band gap, A is a constant depending on the transition probability, *n* depends on the nature of the transition, i.e., allowed direct, allowed indirect, forbidden direct and forbidden indirect. In our case, for an indirect band gap, the value of *n* is ½ [17].

**Figure 4:**
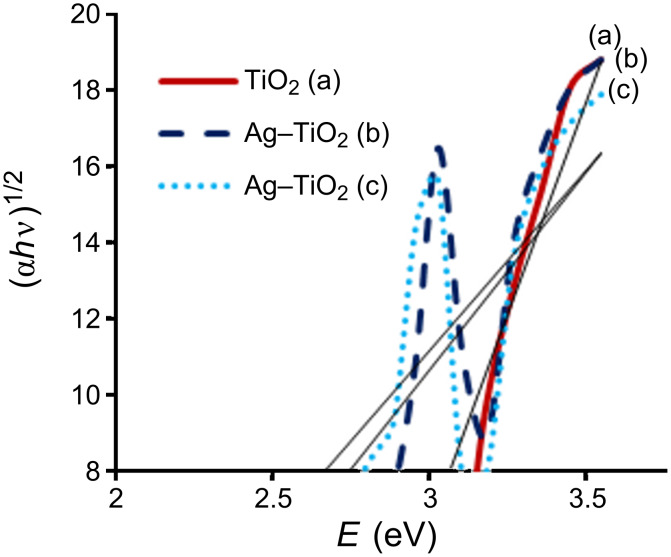
Band gap obtained by extrapolating the linear portion of the (α*h*ν)^1/2^ versus photon energy (eV) curve of (a) TiO_2_ and (b) 3% and (c) 7% Ag-doped TiO_2_ nanoparticles.

The variation of (α*h*ν)^1/2^ with photon energy is shown in Figure 4. The band gaps were determined to be about 3.15 eV, 2.8 eV and 2.7 eV for annealed TiO_2_, Ag–TiO_2_ (3%) and Ag–TiO_2_ (7%), respectively, by extrapolation of the linear portion of the absorption coeffiecient α to zero for indirect-band-gap nanoparticles. The obtained band-gap energy for indirect allowed transitions is in good harmony with the previously reported values [18]. The optical band-gap energies decrease with the doping of silver ions, which allow the delay in recombination rate and enhance the photocatalytic activity.

### Photoluminescence spectroscopy (PL)

Photoluminescence spectroscopy (PL) is a practical method for probing the electronic structure of nanomaterials, the transfer behaviour of the photoexcited electron–hole pairs in semiconductors, and the rate of recombination [19].

**Figure 5:**
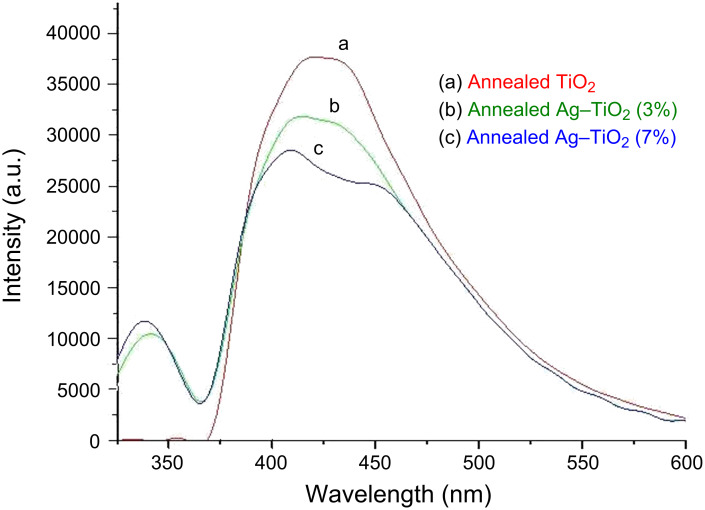
Photoluminescence spectra of annealed TiO_2_ (a) and 3% and 7% Ag-doped TiO_2_ nanoparticles (b,c).

The PL emission spectra of the TiO_2_ and Ag-doped TiO_2_ excited at a wavelength of 285 nm at room temperature are shown in Figure 5. TiO_2_ nanoparticles showed the emission peak in a range of 410–430 nm whereas after doping with silver ions one peak at 333 nm and another one in the range of 400–470 nm appeared in the spectrum. The peaks appearing in the case of Ag-doped TiO_2_ (Figure 5b,c) correspond to the radiative transition of the excited electrons from occupied d bands to higher states of the Fermi level. The PL intensities of annealed Ag-doped TiO_2_ nanoparticles (3% and 7%) were lower in comparison to those of the TiO_2_ nanoparticles because the metallic silver ions cause some changes in the electronic structure of the Ag-containing titanium dioxide nanoparticles [20]. Moreover, the PL intensity of Ag-doped TiO_2_ (7%) is lower in comparison to the case of 3% doping of Ag, and this can be explain as an increased molarity of the silver ions present in Ag–TiO_2_. The PL emission is directly related to the recombination of excited electrons and holes, so the lower PL intensity indicates a delay in recombination rate and, thus, higher photocatalytic activity.

### Antimicrobial activity of TiO_2_ (doped and undoped) nanoparticles

**Figure 6:**
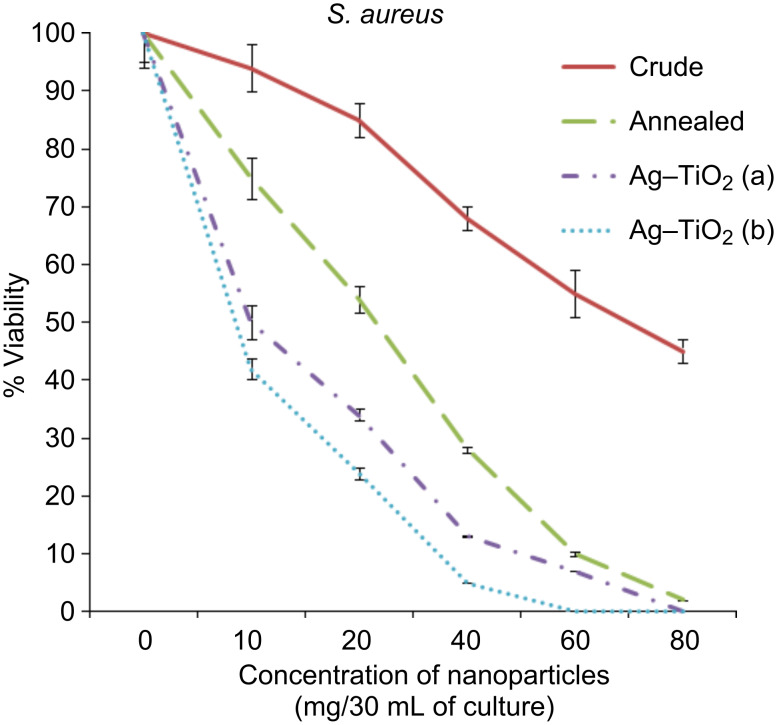
Viability of bacteria (*S. aureus*) against the concentration of nanoparticles (mg/30 mL of culture) in %.

**Figure 7:**
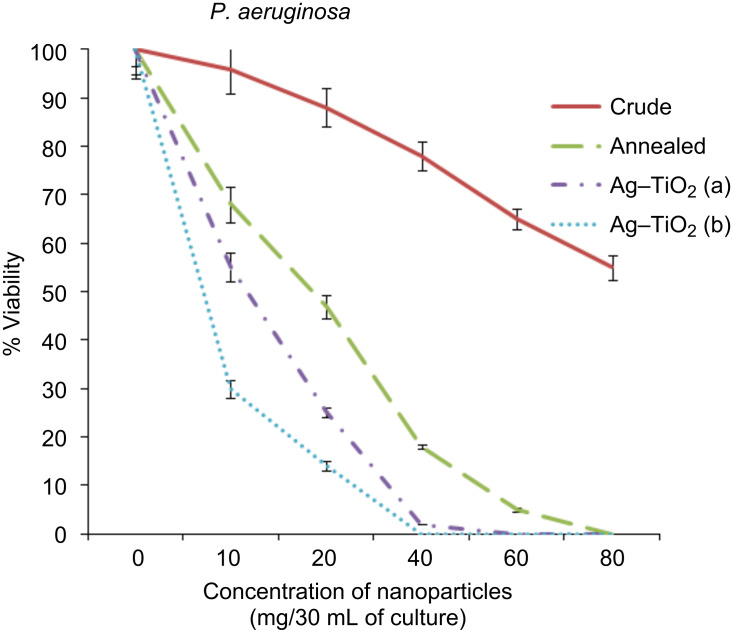
Viability of bacteria (*P. aeruginosa*) against the concentration of nanoparticles (mg/30 mL of culture) in %.

**Figure 8:**
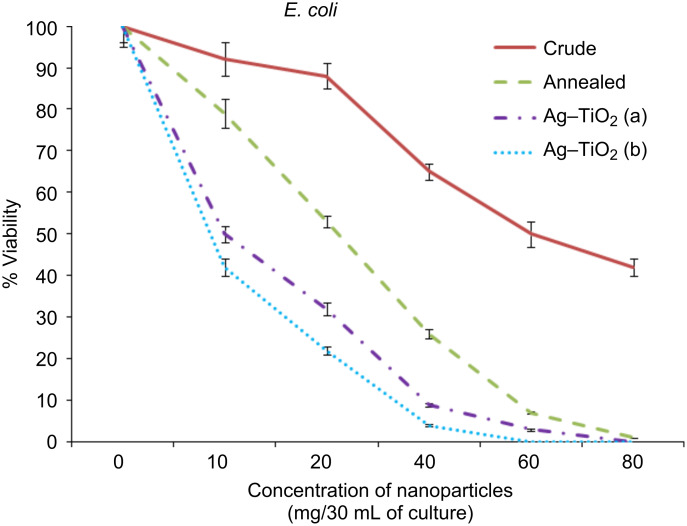
Viability of bacteria (*E.coli*) against the concentration of nanoparticles (mg/30 mL of culture) in %.

The bactericidal activity of the crude and annealed samples of TiO_2_ and Ag-doped TiO_2_ (3% and 7%) were investigated against Gram (+ve) and Gram (−ve) bacteria, as presented in Figure 6, Figure 7 and Figure 8. At 60 mg/30 mL of the culture, silver-doped nanoparticles at both concentrations (3% and 7%) were toxic to all the bacteria tested. However, application of 7% doped Ag–TiO_2_ nanoparticles killed 100% *P. aeruginosa* cells at 40 mg/30 mL concentration, while 5% and 4% viabilities of *S. aureus* and *E. coli* were obtained, respectively. It is also clear from Figure 6, Figure 7 and Figure 8 that crude TiO_2_ nanoparticles showed 45%, 55% and 58% viability loss respectively at 80 mg/30 mL culture concentration, while the same concentration of annealed nanoparticles in culture showed almost 100% viability loss in all three bacterial strains. In the case of 3% silver-doped nanoparticles at 60 mg/30 mL of culture, 0% viability in the case of *P. aeruginosa* was recorded, while in the case of *S. aureus* and *E. coli* 7% and 3% viabilities were recorded. Therefore 7% doped silver nanoparticles at 60 mg/30 mL of bacterial culture (0.2 O.D. at 660 nm) is the optimum concentration for the killing of the bacteria investigated here.

The antibacterial activity of annealed samples is slightly more than crude TiO_2_, because after annealing at 450 °C the amorphous phase of the nanoparticle is converted to both anatase and rutile phases, and shows an indirect band gap of 3.2 eV, which is similar to our result shown in Figure 4 [7]. Due to the indirect and wide band gap of anatase phases present in the annealed sample of TiO_2_, it shows more antibacterial activity than crude TiO_2_. However, the pure TiO_2_ (crude and annealed) nanoparticle showed less photocatalytic activity, while doping of silver ions improves the efficiency under visible-light irradiation. The antibacterial activity of silver-doped TiO_2_ nanoparticles is significantly high in comparison to TiO_2_ nanoparticles. The Ag-doped TiO_2_ showed more photocatalytic activity on Gram-negative bacteria because Gram-positive bacteria have more peptidoglycan than Gram-negative in the cell wall, which is negatively charged, and more silver ions may get trapped to peptidoglycan in Gram-positive bacteria [21]. It is seen that the percentage of viable bacteria exponentially reduced with respect to the increasing concentration of Ag doped into the TiO_2_ matrix.

The results observed from this study, alongside a previous study [22] , suggest that the doping of metal and metal oxides on the surface of TiO_2_ nanoparticles increases the value of the e^−^–h^+^ charge separation by decreasing the band-gap energy, and leads to a delay in the recombination rate and good antibacterial activity (Figure 6–8). The maximum phototcatalytic activity was observed in the case of 7% doping of Ag in the TiO_2_ matrix due to the decreased band-gap energy in comparison to other prepared nanoparticles.

## Conclusion

In this paper we have reported the structural, optical and photocatalytic properties of TiO_2_ and Ag-doped TiO_2_ synthesized by an acid-catalyzed sol–gel technique. The XRD pattern showed the coexistence of silver and anatase TiO_2_ phases. UV–vis spectroscopy revealed the shifting of the absorption edge of silver-doped TiO_2_ to the visible region (higher wavelength) compared to that of the pure TiO_2_ nanoparticles. The PL spectra revealed the structural modification of the TiO_2_ matrix with doping by silver ions, as well as the change in the charge-transfer processes and the delay in the recombination of electron–hole pairs on the surface of TiO_2_. The photocatalysis efficiency of TiO_2_ and Ag-doped TiO_2_ was tested by the percentage viability reduction of bacterial colonies under visible-light irradiation. The viability of *P. aeruginosa* was reduced to zero at 40 mg/30 mL culture of Ag-doped TiO_2_ (7%) while *S. aureus* and *E. coli* showed zero viability at 60 mg/30 mL culture. In the case of 3% doping all bacterial culture were killed at 80 mg/30 mL culture. The pure TiO_2_ (crude and annealed) nanoparticles showed poor photocatalytic activity, while doping of silver ions improves the efficiency under visible-light irradiation.

## Experimental

### Chemicals

Titanium(IV) tetrabutoxide was purchased from Sigma Aldrich, AgNO_3_ from Fluka, and aerosol-OT (AOT) from Sigma Aldrich.

### Microorganisms and culture conditions

Locally isolated log-phase cultures of *S. aureus* (Gram +ve)*, P. aeruginosa* (Gram −ve) and *E. coli* (Gram −ve) were used for analysis of the antimicrobial assay. These cultures were grown in Luria broth and maintained on Luria agar plates. Cultures were grown with 2% inoculums at 25 °C until 0.2 optical density (O.D.) at 660 nm was achieved and used for antibacterial activity.

### Preparation of TiO_2_ nanoparticles

Titanium dioxide nanoparticles were synthesized by an acid-catalyzed sol–gel process [23] starting from titanium(IV) tetrabutoxide (2.94 mM) and using 5 mL of water (pH 2) in the presence of toluene as solvent containing 1% aerosol-OT (reverse micelles) under stirring for 1 h:


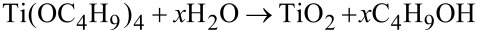


After gelation, the gel was dried at 100 °C in an oven for 24 h; white TiO_2_ nanosized particles (crude sample) were obtained. To obtain crystalline particles, samples were annealed at 450 °C for 30 min.

#### Preparation of Ag-doped TiO_2_ nanoparticles

Ag-doped titanium dioxide nanoparticles were synthesized by using an acid-catalyzed sol–gel process starting from titanium(IV) tetrabutoxide (2.94 mM) using 5 mL of water (pH 2) in presence of toluene as solvent containing 1% aerosol-OT (reverse micelles). The appropriate concentration of silver salt (3% or 7%) in 0.5 mL deionized water was dropwise added to the reaction mixture under stirring. After gelation, the nanoparticles were allowed to dry in an oven at 100 °C for 24 h to give a white powder. This was subjected to further heat treatment at 450 °C for 30 min. Upon heating to 450 °C, silver-doped materials showed a discernible colour change of the starting powder from white to grey.

#### Suspension of TiO_2_ and Ag-doped TiO_2_ nanoparticles

1% suspension of TiO_2_ and Ag-doped TiO_2_ of nanoparticles were prepared in 0.8 M NaCl.

#### Characterization of TiO_2_ and Ag-doped TiO_2_ nanoparticles

The XRD scan of the nanoparticles was performed on a Tecnai 20 G^2^ X-ray diffractometer by using Cu Kα radiation (FEI). The samples were scanned over a range of 5–70°. The transmission electron microscopy was done on a JEOL JEM-1011. UV–vis spectra of the samples were taken on a LABOMED UV–vis spectrometer from 200 to 1000 nm. Photoluminescence spectra was recorded on an Ocean Optics system with a range of 325–640 nm, by using an excitation wavelength of 285 nm and slit width of 2 nm.

### Assay for the antibacterial activity of TiO_2_ and Ag-doped TiO_2_ against *S. aureus*, *P. aeruginosa* and *E. coli*

The photocatalytic activities of TiO_2_ and Ag-doped TiO_2_ matrices were evaluated against *S. aureus*, *P. aeruginosa* and *E. coli* under visible light at room temperature (25 ± 2 °C) by % viability (% survival) of bacteria using different concentrations of doped and undoped nanoparticles. The prepared suspensions of nanoparticles (1%) were used in different concentrations, i.e., 10, 20, 40, 60 and 80 mg in 30 mL of bacterial culture having 0.2 O.D. at 660 nm and stirred for 4 h in the presence of fluorescent light having a light intensity of 500 lux. The mixture of nanoparticles and bacterial culture was spread over Luria agar plates, and the viability of the bacterial cell was checked by its colony-forming ability.
