# Correction: Photocatalytic antibacterial performance of TiO_2_ and Ag-doped TiO_2_ against *S. aureus. P. aeruginosa* and *E. coli*

**DOI:** 10.3762/bjnano.11.43

**Published:** 2020-04-03

**Authors:** Kiran Gupta, R P Singh, Ashutosh Pandey, Anjana Pandey

**Affiliations:** 1 Department of Chemistry, Motilal Nehru National Institute of Technology, Allahabad, Prayagraj, 211004, U.P., India; 2 Nanotechnology & Molecular Biology Lab, Centre of Biotechnology, University of Allahabad, Allahabad, Prayagraj, 211002, U.P., India; 3 Department of Biotechnology, Motilal Nehru National Institute of Technology, Allahabad, Prayagraj, 211004, U.P., India

**Keywords:** Ag-doped TiO_2_, antimicrobial activity, sol–gel

The following is a correction to the section “XRD of TiO_2_ and Ag-doped TiO_2_”, which contains a new Figure 1, an analysis of the data in the new Figure 1, and the raw data files used to create Figure 1 (included as Supporting Information File 1). This correction is being issued in response to questions raised regarding the originally published experimental results in Figure 1. This issue was investigated further by the authors who were able to retrieve the correct raw data files associated with their materials. Although significantly different from the originally published, incorrect experimental data, the interpretation of the new, correct data does not affect any other sections or any other results of the article.

## XRD of TiO_2_ and Ag-doped TiO_2_ nanoparticles

X-ray diffraction (XRD) was used to characterize as-prepared TiO_2_ and Ag-doped TiO_2_ nanoparticles. The diameter of crystalline TiO_2_, 3 wt % Ag-doped TiO_2_ and 7 wt % Ag-doped TiO_2_ nanoparticles annealed at 450 °C was calculated by the Scherrer equation to be approximately 20, 22, and 16 nm, respectively. The analysis was based on the broadening of the (101) XRD peak of the pattern shown in Figure 1a–c.

**Figure 1:**
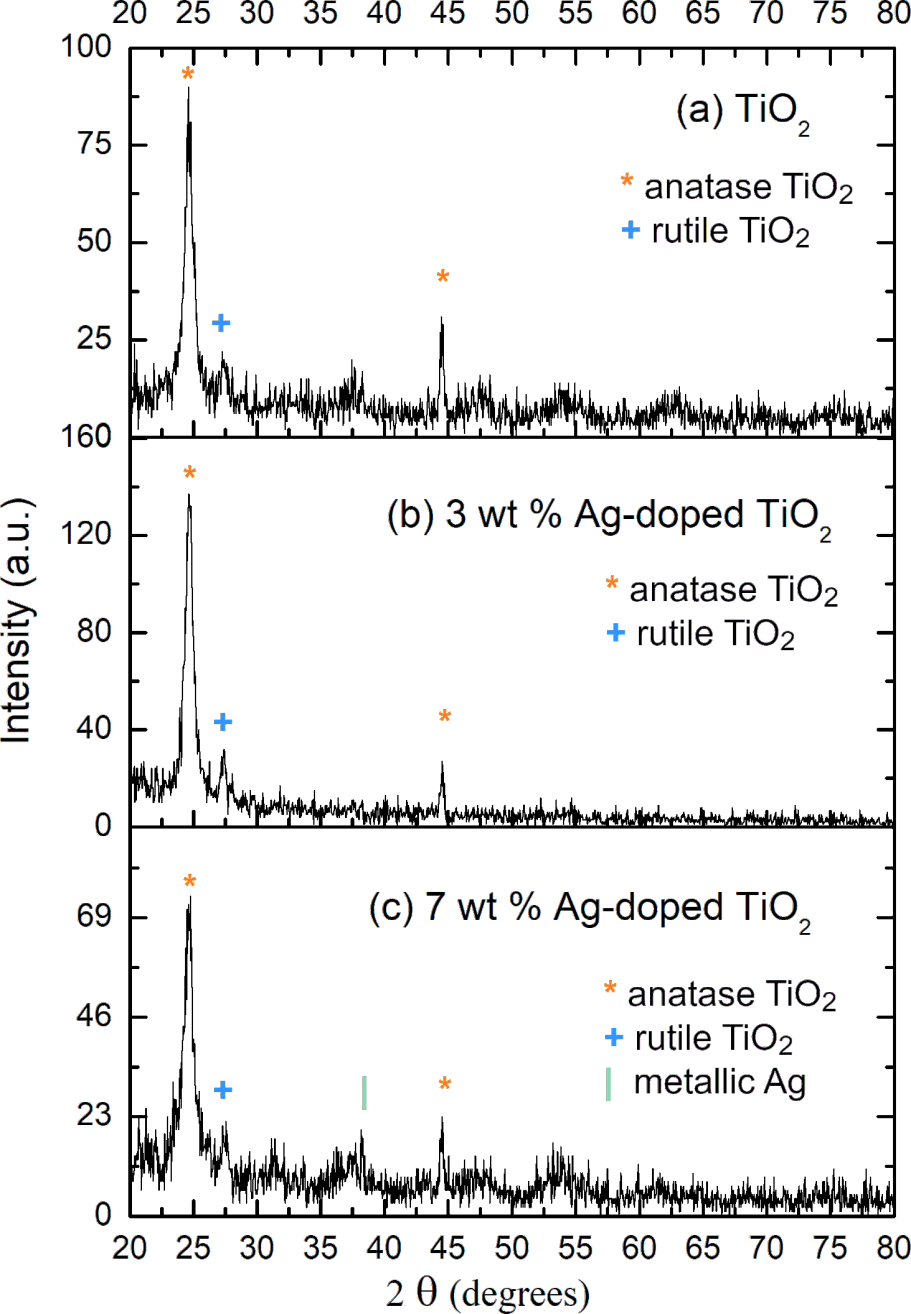
XRD patterns of (a) TiO_2_ nanoparticles, (b) 3 wt % Ag-doped TiO_2_ nanoparticles and (c) 7 wt % Ag-doped TiO_2_ nanoparticles annealed at 450 °C. Supporting Information File 1 contains the raw data files used to create this figure.

The XRD analysis was performed with X-pert High score plus software to determine the phase structure of the three samples by comparing the measured XRD pattern to powder diffraction patterns in the International Centre for Diffraction Data (ICDD) database. The characteristic peaks of the TiO_2_ nanoparticle sample indicate an anatase phase (2θ = 24.8°, 44.5°, compared with JCPDS file no. 00-021-1272) with some indication of a rutile phase (2θ = 27.5°, compared with JCPDS file no. 00-021-1276), revealing the effect of calcination.

As expected from previous works on similar Ag-doped TiO_2_ nanoparticles [1–5], the diffraction peaks associated with Ag were not easily observed. Similar to these prior works, the presence of Ag did not cause changes in the TiO_2_ anatase crystalline structure and no significant high intensity peaks related to fcc Ag were observed. This can be explained as the mean silver peak can be masked by the TiO_2_ layer.

However, by comparing the ratio of the intensity of the peaks, we can conclude that a peak at 2θ **≈** 38°, indicating the presence of Ag (compared to JCPDS file no. 00-0004-0783), was weak but not absent for the 7 wt % Ag-doped TiO_2_ nanoparticle sample. This result corresponds to a prior work where it was reported that 3.5 wt % Ag-doped TiO_2_ calcined at 500 °C did not show any peaks relating to Ag, although very low intensity peaks related to Ag were observed for the sample calcined at 600 °C [6].

In a previous work, it was found that the intensity of the anatase peaks decreased in comparison to the rutile peaks as the annealing temperature increased; and after annealing at 800 °C, complete rutile TiO_2_ phase was obtained [7]. It was previously reported that a mixture of anatase and rutile TiO_2_ nanoparticles has higher photocatalytic activity than pure anatase or pure rutile TiO_2_ nanoparticles under UV-light excitation [8]. Furthermore, it was shown that calcination of the nanoparticles could increase the crystallinity of TiO_2_, which leads to a decrease in the photo-excited e^−^ –h^+^ recombination, and thus, to an increase in the photocatalytic activity of TiO_2_ [9].

## Supporting Information

A ZIP file containing the raw data files associated with the three XRD patterns presented in Figure 1.

File 1 Raw data files.
